# Conversion Practices in Eating Disorder Treatment: A Lived Experience Narrative

**DOI:** 10.1111/jpm.70168

**Published:** 2026-06-23

**Authors:** Cody Spadaccini, Kai Schweizer

**Affiliations:** ^1^ Independent Researcher and Lived Experience Advocate Western Australia Australia; ^2^ School of Human Sciences (Exercise & Sport Science) University of Western Australia Perth Western Australia Australia; ^3^ The Kids Research Institute Australia Perth Western Australia Australia

## Abstract

**Background and Aim:**

Conversion practices (sometimes referred to as ‘conversion therapy’) refer to interventions that aim to change a person's sexual orientation or gender identity. While these are often associated with faith‐based settings, they can also occur in secular healthcare contexts. The practices are associated with significant psychological harm. This paper aims to provide a lived experience account of gender identity conversion practices perpetrated in the context of inpatient eating disorder (ED) treatment.

**Methods:**

This paper provides the first author's narrative of his inpatient ED treatment as a young trans man and maps this experience to the key features aligned with accepted definitions of conversion practices and their enduring impact.

**Findings:**

This paper describes an inpatient ED treatment experience characterised by (1) pathologisation of the first author's gender diversity; (2) the use of coercion and institutional power to enforce cisnormative developmental outcomes; (3) the persistent withholding, delaying, and problematising of gender‐affirming medical care; and (4) the enduring psychological harm and extended disengagement from further treatment‐seeking that results from these practices.

**Discussion:**

This narrative highlights the urgent need to safeguard against conversion practices within inpatient mental health settings and ensure ED treatment is provided from a gender‐affirming framework.

## Accessible Summary

**What is known on the subject?:**

- Conversion practices aim to change or suppress a person's gender identity or sexual orientation and are harmful and unethical.
- In health settings, conversion practices can appear as pathologising identity, identity suppression, gatekeeping or withholding gender affirming care.

**What the paper adds to existing knowledge?:**

- This paper is a lived experience account of conversion practices within inpatient ED treatment.
- It highlights the potentially widespread nature of conversion practices within ED treatment perpetrated against trans and gender‐diverse people

**What are the implications for mental health nursing?:**

- The paper identifies how mental health nurses can prevent conversion practices in ED treatment by affirming a patient's gender identity in screening and treatment.

## 1 Introduction

Conversion practices (otherwise referred to as conversion therapy, reparative therapy, realignment therapy and sexual orientation and gender identity change efforts, ‘SOGICE’) refer to any intervention that aims to change or suppress a person's sexual orientation or gender identity (Anderson et al. 2024). These practices are underpinned by ‘conversion ideology’, which posits that being cisgender and heterosexual is the natural state, and any deviation from this is a sign of brokenness or pathology that needs to be remedied (Jones et al. 2021).

While these practices are typically associated with faith‐based settings, they can also occur in healthcare contexts and be conducted by mental health professionals such as psychologists and psychiatrists (Kinitz et al. 2021). This can include more overt practices such as psychotherapeutic and pharmacological interventions, as well as more covert practices such as the denial or delay of gender‐affirming medical care or the pathologising of trans and gender diverse identities in assessment and treatment (Kinitz et al. 2021).

Within the literature, several key features of conversion practices are consistently described:

- A guiding ideology that frames being cisgender and heterosexual as the healthy, natural state and positions diverse sexual orientations, gender identities and gender expressions as something that should be prevented, suppressed or discouraged.
- Pathologising a diverse sexual orientation or gender identity, including framing it as the symptom of an illness, the result of trauma or a moral failing, rather than a valid form of human diversity.
- Use of coercion and power imbalances in clinical, faith‐based and/or family settings, such that a person cannot meaningfully refuse participation, or withdrawal from the intervention would result in significant negative consequences.
- Enforcing cisgender and heterosexual norms, including through withholding, delaying or problematising gender‐affirming medical care.

Sexual orientation and gender identity are distinct constructs. Sexual orientation refers to a person's sexual and romantic attraction, whereas gender identity refers to a person's internal sense of being a woman, man or another gender. While the broader literature on conversion practices often uses umbrella terms such as SOGICE that include both sexual orientation and gender identity, the present narrative focuses specifically on gender identity conversion practices.

There is no high‐quality evidence that conversion practices can alter a person's gender identity (Anderson et al. 2024). However, there is substantial evidence that these practices can cause significant harm to survivors, including higher rates of depression, anxiety, post‐traumatic stress disorder and suicidality (Anderson et al. 2024; Kinitz et al. 2021).

Conversion practices do not occur in a vacuum; they are shaped by sociocultural representations of gender diversity, as well as by political and institutional contexts that influence how trans identities and gender‐affirming medical care are understood within healthcare. For example, recent restrictions on puberty‐suppressing medications for trans and gender diverse young people in the United Kingdom illustrate how wider sociocultural and political climates contribute to the normalisation, minimalisation, or obscuring of practices that can function as conversion practices within healthcare (Kennedy 2025).

Increasingly, the literature regarding trans and gender diverse experiences of eating disorder (ED) treatment highlights practices that align with accepted definitions of conversion practices (Goetz and Wolk 2023; Joy et al. 2022; Schweizer et al. 2025). This paper presents a lived experience narrative of a survivor of conversion practices perpetrated during ED treatment. It aims to support mental health nursing practice that protects autonomy, avoids identity suppression and delivers safe, gender‐affirming treatment.

## 2 Methods

This paper presents a collaborative lived experience narrative of the first author's experience of conversion practices in ED treatment. The first author (CS) prepared an initial written account of his experiences and shared this with the second author (KS). KS reviewed this account and provided feedback and support with possible conceptual framing, structure, connection to the literature and alignment with journal conventions. CS then revised the written narrative, and the manuscript was developed through an iterative process of refinement. Throughout this process, CS retained authority over the lived experience narrative and ultimate decision‐making over all aspects of the manuscript. KS's role was to support CS in the development of the manuscript into a format suitable for peer‐reviewed publication. As a result of the collaborative nature of this work, the paper is written in third person at the request of CS. However, the narrative presented remains grounded in CS's experiences, emotions and interpretations.

## 3 Lived Experience of Conversion Practices

**FIGURE 1:**
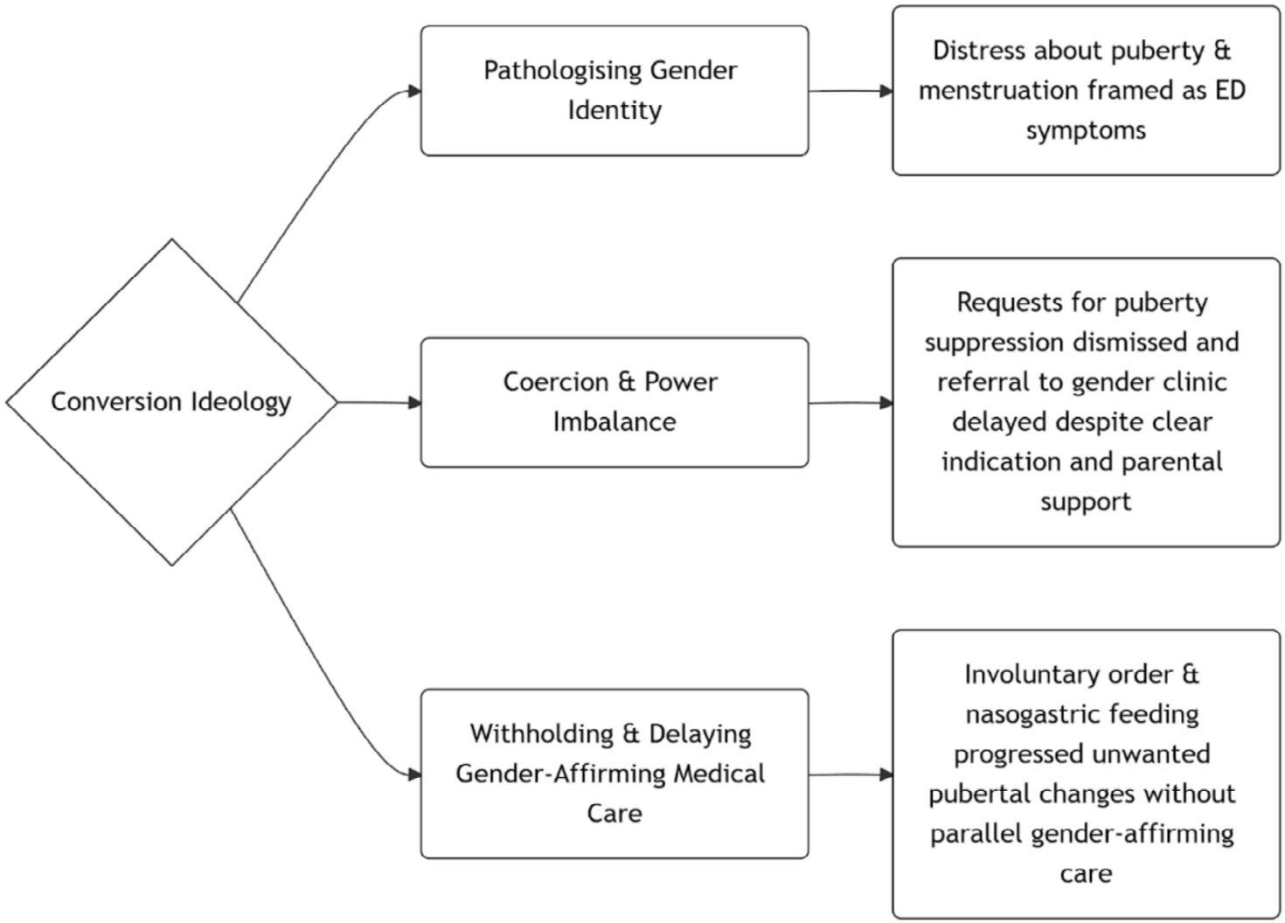
Key features of conversion practices observed in Cody's inpatient eating disorder treatment.

This narrative describes the lived experience of an adult trans man, reflecting on his treatment at age 14 in a paediatric ED unit due to medical instability. Cody's experiences are interpreted as conversion practices not on the basis of any single clinical decision, but rather based upon the cumulative pattern in which his inpatient care repeatedly sought to suppress, eliminate, or change his gender identity. The following results are organised around the key features of conversion practices and illustrate how his experience aligns with them (see Figure 1).

## 4 Pathologising a Diverse Gender Identity

Throughout his hospitalisation, Cody's gender identity was consistently framed as pathological or as a symptom of his ED, rather than acknowledged as a valid expression of gender diversity. In his initial psychiatric evaluation, he disclosed that he had profound distress about developing female secondary sexual characteristics and explicitly stated that his disordered eating behaviours functioned to suppress his puberty. He also expressed a desire to have a mastectomy and hysterectomy in future. The assessing psychiatrist labelled these disclosures as ‘unrealistic’ and ‘overvalued ideas’. No exploration of gender identity or mention of gender dysphoria was offered; his fears and distress were presumed to be delusional or solely a product of malnutrition and mental illness.

His medical notes show that his persistent focus on avoiding puberty was seen as an irrational ‘fear’ to be overcome. In fact, the treating doctor wrote that there was ‘little to be gained by avoiding the object of fear’ (i.e., puberty) and that it was better to force him to face it under supervision. This approach denied the legitimacy of his gender identity, interpreting it as a phobic symptom. Team members suggested that once he gained weight and was no longer malnourished, he would ‘be a normal teenage girl.’ Indeed, clinicians openly told him that his thoughts about not being a girl were simply due to his illness and would disappear with proper nutrition and psychotherapy. However, this proved untrue. After weight restoration, he continued to experience intense gender dysphoria and mental health struggles; his trans identity was not a product of starvation or a temporary phase.

Cody's treating clinicians appeared to be guided by an implicit assumption that cisgender, heterosexual development was the only healthy outcome. For example, when he insisted that he wanted a hysterectomy as soon as he could access one, the doctor responded dismissively, ‘What are we going to do when you're twenty‐five and want to get a husband and have kids?’ This remark assumed a heteronormative, cisgender future for him, implying that his current gender identity and acute gender dysphoria about menstruation were not real and would eventually resolve into a desire for a conventional female life. Such comments reflected a conversion ideology: the doctor presumed his feelings would resolve with treatment and he would revert to a ‘normal’ cisgender woman in adulthood, positioning his current identity and wishes as a misguided pathology to be corrected.

## 5 Use of Coercion and Power Imbalances

Cody's experience of ED treatment was characterised by a pronounced power imbalance, where he was unable to refuse interventions within a context where cisnormative outcomes were enforced. At admission, when Cody disclosed the extent of his distress about puberty, including the intent to end his life if he menstruated, his treating team immediately placed him on an involuntary treatment order. As a result, he had no agency to decline treatment or leave the hospital. He was a minor confined to a clinical setting where adults with medical authority controlled his care. His subsequent attempts to communicate his needs verbally and in written letters were dismissed and did not alter his treatment plan. Cody was clear with his clinicians that his ED functioned to delay pubertal changes. This presentation required a gender‐affirming treatment protocol that integrated gender‐affirming medical care, so that his ED behaviours no longer needed to serve their protective function.

Instead, his treatment team used restrictive practices to involuntarily insert a nasogastric tube. While nasogastric feeding is commonly used in inpatient ED settings, in the context of unaddressed gender dysphoria, this was not a neutral medical decision. It was a coercive pathway that enforced the progression of female puberty. Despite clear statements by Cody that pubertal progression would elevate his suicidality and distress, his treatment team utilised their institutional power to enforce a cisnormative development outcome, while denying access to gender‐affirming medical care that would have addressed the underlying function of his ED, alleviated his gender dysphoria and minimised his suicidality.

## 6 Withholding, Delaying and Problematising Gender‐Affirming Medical Care

A defining feature of Cody's experience was the withholding and delaying of gender‐affirming care, as well as framing such care as illegitimate or inappropriate. Despite his clear and repeated expressions that the function of his ED was to prevent female puberty, the clinical team refused to provide interventions that could have suppressed his puberty in a safe, monitored manner. The most glaring example of this was that, early in his admission, he directly inquired about puberty blocking medication. Rather than consider or discuss this option, the doctor immediately shut down the conversation, telling him, ‘You didn't hear that from me,’ and refusing to entertain the topic further. This response effectively withheld potentially lifesaving medical information and treatment. Puberty blockers are a standard care option for trans adolescents, and are even routinely used for cisgender children with precocious puberty. Yet for him, the doctors treated the idea of suppressing puberty as unspeakable.

Additionally, clinicians used their institutional authority to undermine his mother's attempts to advocate for medical interventions that could delay his puberty or suppress menstruation. Over the course of his admission, the treating team persuaded Cody's mother that gender‐affirming medical care options were not in his best interest, framing them as unsafe or inappropriate. This eroded parental advocacy and further discouraged and delayed access to gender‐affirming medical care. This positioned gender‐affirming medical care as an illegitimate pathway and contributed to delayed and denied access to care that could have addressed the underlying function of Cody's ED and alleviated his gender dysphoria.

The care team also significantly delayed any form of gender affirmation or options to alleviate gender dysphoria until very late into treatment. Towards the end of his hospital admission, during which weight restoration resulted in pubertal progression, his treating clinicians finally referred him to a gynaecologist. The gynaecologist argued that he must go through one menstrual cycle ‘for the sake of bone health,’ then he could be prescribed the oral contraceptive pill to suppress further periods. While this care at least validated his distress, it still required him first to experience the very event he had described as intolerable and suicidality‐inducing. The insistence on a mandatory menstrual cycle (and the concern about bone density) exemplifies how providers problematized gender‐affirming care, treating it as dangerous or unjustified and prioritised other considerations over his urgent psychological needs. In the end, even this compromise failed him: the birth control method did not fully prevent menstruation, leading to repeated breakthrough bleeding that exacerbated his depression and trauma. In hindsight, access to puberty blocking medication could have prevented significant harm, but this option was continually withheld.

Referrals to appropriate gender specialists were also delayed or never made. The children's hospital where he was treated had a gender clinic with professionals experienced in supporting trans youth. However, he was not informed of its existence until his discharge was imminent. By that point, he was so traumatised and distrustful of medical professionals that he could not imagine pursuing further help. In essence, the opportunity for him to receive specialised gender‐affirming support when requested was denied, robbing him of the chance for validation and guidance when he needed it most. Instead, the team persisted in viewing and treating him solely as an ED patient. The overall effect was that every possible avenue of gender affirmation (hormonal, surgical and even social support) was blocked or postponed, while the natural progression of female puberty was actively forced upon him. This systematic withholding of appropriate care and insistence on a cisgender outcome are hallmarks of conversion practices in a medical context.

## 7 Psychological Harm and Disengagement From Healthcare

The psychological harm associated with these conversion practices was profound and enduring. Following discharge, Cody had a marked deterioration in his mental health. Rather than resolving his ED or alleviating his distress, the admission exacerbated his ED and left him traumatised and hopeless. He felt as though a part of him had ‘died’ during his inpatient hospitalisation. After being forced to undergo irreversible pubertal changes during his admission, Cody experienced profound despair and identity fragmentation. His body continued to develop in ways that felt alien to him. He became socially withdrawn, spending long periods confined to his bedroom and developed agoraphobia.

While the inpatient admission may have restored Cody's medical stability, it did not address the underlying driver of his distress, leaving him with ongoing disordered eating and significant psychological suffering. His ED did not resolve but instead shifted to binge eating and purging behaviours more consistent with bulimia nervosa to manage his ongoing distress, unaddressed gender dysphoria and trauma.

Cody also experienced a profound loss of trust in healthcare professionals. He felt unseen and invalidated by those responsible for his care, and this contributed to long‐term avoidance of medical settings. When he had to interact with healthcare providers (e.g., for routine checkups or acute illnesses), he experienced acute responses, including uncontrollable shaking. In the years that followed his admission, he did not pursue follow‐up care for ED recovery, nor gender‐affirming medical care, because he anticipated, based on his previous experience, that he would not be believed, listened to and would endure further harm. For almost a decade, Cody attempted to self‐manage his gender dysphoria, depression, post‐traumatic stress disorder and ED.

## 8 Recovery

After almost a decade of disengagement with healthcare services, Cody re‐engaged in his mid‐twenties. A trusted friend had recommended a specific general practitioner who took a gender‐affirming approach. Even with this endorsement, re‐entering medical settings required substantial courage due to his previous experiences of conversion practices. With the support of this general practitioner, Cody commenced testosterone therapy and later underwent gender‐affirming chest ‘top’ surgery. These interventions, which his ED clinicians had withheld and framed as inappropriate, were profoundly beneficial to him. He recalls the days he received his testosterone prescription and the day he woke up from his top surgery as the happiest and most affirming experiences of his life. With these interventions, his mental health and quality of life improved significantly. For the first time in his life, he was able to imagine a future for himself.

However, these positive outcomes were severely delayed. Cody continues to live with the psychological consequences of being a conversion practices survivor. He experiences ongoing complex post‐traumatic stress disorder attributed to medical trauma and identity suppression, and his ED has remained an ongoing issue requiring continued management. In reflecting on this trajectory, Cody's account illustrates how conversion practices in healthcare settings can produce harm that extends well beyond the period of treatment. The practices he experienced did not change his gender identity, but they did contribute to prolonged suffering and delayed access to care that ultimately improved his well‐being. His ED continues to require ongoing monitoring and management.

## 9 Preventing Conversion Practices in Eating Disorder Treatment: A Call to Action

Cody's experience of treatment occurred in 2012, when professional understanding of gender diversity and its intersection with EDs was less developed than it is today. However, similar experiences have been described in the past 5 years in Australia (Schweizer et al. 2025), the United States (Goetz and Wolk 2023) and Canada (Joy et al. 2022).

The publication of this narrative in an academic nursing journal is intentional. Lived experience accounts can illuminate forms of harm that are not easily captured via research, clinical documentation or psychometric instruments. This is especially relevant to conversion practices, which are sometimes obscured when they become normalised and embedded within treatment. With this context in mind, Cody's story is intended to contribute to education, advocacy, safeguarding and practice change within the field of mental health nursing. It is hoped that this account will support critical reflection among mental health nurses and assist in identifying practices that may function as conversion practices.

Cody's experience of conversion practices did not change his identity, but it caused substantial psychological harm and contributed to prolonged disengagement from healthcare. His story underscores the urgent need for ED treatment that is gender‐affirming, trauma‐informed and respects autonomy.

As an individual lived experience narrative, Cody's experience does not represent the experiences of all trans and gender diverse people with ED treatment. Instead, this paper provides an in‐depth account of one person's experience to highlight how conversion practices may be enacted and experienced within a clinical setting. Mental health nurses are uniquely placed to prevent conversion practices and intervene when they occur in inpatient settings, as they often have the most frequent and sustained engagement with patients.

Mental health nurses are well‐placed to protect the dignity and safety of trans patients in inpatient ED settings, as they often provide continuous care and have the most interaction with the patient. This position creates repeated opportunities to build trust, recognise harm and foster care that affirms rather than suppresses identity. Central to this role is recognising gender diversity as a valid form of human diversity and ensuring that treatment formulations do not pathologise gender diversity or conflate gender dysphoria with body image disturbances such as body dysmorphic disorder. Nurses can further support inclusive care that does not presume a cisnormative or heteronormative life trajectory, but instead reflects the diversity of genders, bodies and futures. In practice, this means ensuring that treatment environments are gender‐affirming not only in patient‐facing materials, but also in everyday interactions and the broader service culture.

Nurses can also create conditions that make disclosing a diverse gender identity feel safe. This safety can be fostered through practices such as asking patients their name and pronouns at intake, ensuring systems can document both legal and chosen names and using affirmed name and pronouns consistently across interactions whenever it is safe to do so. Affirming practice also involves supporting patient‐led communication by asking who is aware of a person's gender identity, what language is safe in different contexts, and how information should be shared with family members, carers and staff. Opportunities for private discussion should be routinely provided, particularly for young people, and confidentiality should be clearly explained. Where patients do not yet have the language, safety or confidence to describe their experiences directly, nurses can create repeated, low‐pressure opportunities for disclosure over time. These communication practices are most effective when they are embedded within service‐wide norms that support pronoun sharing, respect privacy and promote consistency across staff and shifts.

Mental health nurses can help ensure that assessment and treatment planning are responsive to the meanings and functions of ED behaviours. When a patient describes distress related to puberty, menstruation or secondary sex characteristics, this should be met with curiosity, validation and clinical attention. Where disordered eating functions to reduce gender dysphoria or suppress puberty, formulation and care planning should account for the possibility that refeeding and weight restoration may intensify gender dysphoria and suicide risk. Within this context, gender‐affirming medical care may be an important component of safe treatment, rather than something peripheral to ED care. Nurses can help to reduce distress and strengthen treatment engagement with timely referrals to relevant medical providers as well as peer and community support. They can also advocate for structured staff education in trans health and gender‐affirming ED care so that these issues are recognised and addressed with greater competence across the multidisciplinary team.

Within restrictive legal or institutional contexts, nursing practice remains central to protecting and maximising patient autonomy. This includes communicating treatment plans directly with patients, inviting their preferences and pursuing the least restrictive approach consistent with medical safety. Where restrictive or coercive interventions are required, these should be approached as significant clinical events requiring clear rationale, documentation, debriefing and active harm minimisation. Nurses can also contribute to accountability by identifying practices that may function as conversion practices, documenting concerns and escalating these through appropriate clinical leadership and reporting pathways. Beyond individual cases, nurses can contribute to broader advocacy by challenging oppressive systems and practices, supporting institutional and professional cultures that are gender‐affirming, and participating in collective efforts to resist policies and practices that enable conversion practices within healthcare. This can include supporting workforce diversity, advocating for the inclusion of trans staff and peer workers and strengthening lived experience capacity within services.

## 10 Relevance Statement

Mental health nurses are central to the safety, quality and ethical integrity of inpatient eating disorder care. This lived experience narrative illustrates how conversion practices can occur in healthcare settings through the pathologisation of gender diversity, coercive treatment and the delaying or withholding of gender‐affirming medical care. By describing the enduring psychological harms associated with these practices, it highlights the importance of nursing practices that safeguard patient autonomy and promote gender‐affirming, trauma‐informed eating disorder treatment for trans and gender diverse people.

## 11 Cody's Final Remarks

It is my hope that in sharing my story I can help to bring awareness to the subject of conversion practices in medical settings. To help psychiatric nurses and other medical professionals gain an understanding of what a patient in similar circumstances may be feeling and to be aware of the consequences of non‐affirming care.

I feel that my experience broke me down to the point that I gave up advocating for help. This led to over a decade of self‐managing severe mental health challenges; I felt like a shell of a person and experienced chronic suicidal ideation, resulting in a serious attempt that I survived against all odds.

Over the past few years, I have begun the process of healing through re‐engaging with doctors and mental health professionals. I hold a deep sense of gratitude for the people that have supported me during this time. I can see that there has been significant positive progress in my life, which I attribute to their help.

I wonder how my life may have taken a different course if I had felt seen, heard and believed as a young teenager. If I was treated with dignity and respect, and given access to the care I was so desperately trying to advocate for. We have the opportunity now to shape a new standard of care, to safeguard young people from encountering anything akin to my experience.

There is a long way to go to create a greater understanding of trans and gender diverse presentations within the medical field and society as a whole. Through increased education and people sharing their lived experience, I hope that we can begin to see real change to eradicate conversion practices.

## Conflicts of Interest

The authors declare no conflicts of interest.

## Data Availability

Data sharing not applicable to this article as no datasets were generated or analysed during the current study.

## References

[jpm70168-bib-0001] Anderson, J. R. , T. W. Jones , J. Power , et al. 2024. “Engaging Mental Health Service Providers to Recognize and Support Conversion Practice Survivors Through Their Journey to Recovery.” Cognitive and Behavioral Practice 31, no. 1: 20–25. 10.1016/j.cbpra.2023.08.005.

[jpm70168-bib-0002] Goetz, T. G. , and C. B. Wolk . 2023. “Moving Toward Targeted Eating Disorder Care for Transgender, Non‐Binary, and Gender Expansive Patients in the United States.” International Journal of Eating Disorders 56, no. 12: 2210–2222. 10.1002/eat.24055. 37638738 PMC11773632

[jpm70168-bib-0003] Jones, T. , T. W. Jones , J. Power , M. Pallotta‐Chiarolli , and N. Despott . 2021. “Mis‐Education of Australian Youth: Exposure to LGBTQA+ Conversion Ideology and Practises.” Sex Education 22, no. 5: 595–610. 10.1080/14681811.2021.1978964.

[jpm70168-bib-0004] Joy, P. , M. White , and S. Jones . 2022. “Exploring the Influence of Gender Dysphoria in Eating Disorders Among Gender Diverse Individuals.” Nutrition & Dietetics 79, no. 3: 390–399. 10.1111/1747-0080.12727. 35231954

[jpm70168-bib-0005] Kennedy, N. 2025. “Harming Children: The Effects of the UK Puberty Blocker Ban.” Journal of Gender Studies: 1–17. 10.1080/09589236.2025.2521699.

[jpm70168-bib-0006] Kinitz, D. J. , T. Goodyear , E. Dromer , et al. 2021. ““Conversion Therapy” Experiences in Their Social Contexts: A Qualitative Study of Sexual Orientation and Gender Identity and Expression Change Efforts in Canada.” Canadian Journal of Psychiatry 67, no. 6: 441–451. 10.1177/07067437211030498. 34242106 PMC9152241

[jpm70168-bib-0007] Schweizer, K. , F. Austin , K. Wright , et al. 2025. “Eating and Exercise Experiences of Australian Trans and Gender Diverse Folks: Lived Experience and Stakeholder Perspectives.” International Journal of Transgender Health 27: 1–24. 10.1080/26895269.2024.2447765.42619653

